# Alternative Growth and Defensive Strategies Reveal Potential and Gender Specific Trade-Offs in Dioecious Plants *Salix paraplesia* to Nutrient Availability

**DOI:** 10.3389/fpls.2016.01064

**Published:** 2016-07-20

**Authors:** Hao Jiang, Sheng Zhang, Yanbao Lei, Gang Xu, Dan Zhang

**Affiliations:** ^1^Key Laboratory of Mountain Surface Processes and Ecological Regulation, Institute of Mountain Hazards and Environment, Chinese Academy of SciencesChengdu, China; ^2^School of Life Sciences, Southwest University of Science and TechnologyMianyang, China

**Keywords:** desertification, dioecy, low soil fertility, sex differences, willow

## Abstract

Population sex ratios of many dioecious plants in nature are biased. This may be attributed to sexually different resource demands and adaptive capacity. In male-biased*Populus*, males often display stronger physiological adaptation than females. Interestingly, *Populus* and *Salix*, belonging to Salicaceae, display an opposite biased sex ratio, especially in nutrient-poor environmental conditions. Do female willows have a greater tolerance to nutrient deficiency than males? In this study, we investigated the growth and defensive strategies of *Salix paraplesia* cuttings, which were grown with high and low soil fertility for about 140 days over one growing season. Results suggest that different strategies for biomass allocation may result in sexually different defense capacities and trade-offs between growth and defense. Females are likely to adopt radical strategies, overdrawing on available resources to satisfy both growth and defense, which seems to be more like a gamble compared with males. It is also suggested that females may have an extra mechanism to compensate for the investment in growth under nutrient-poor conditions. In summary, the results may help focus restoration efforts on sex selection such that a moderate increase in female willow quantity could increase the resistance and resilience of willow populations to early sporadic desertification.

## Introduction

Dioecy is found in 175 flowering plant families and in 7% of flowering plant genera (Renner, 2014). Classic sex ratio theory suggests that when reproductive costs to produce a female vs. a male offspring are equal, natural selection will act to balance the sex ratio of the population (Fisher, 1930). However, numerous investigations have revealed that the population sex ratios of many dioecious plants are biased (Barrett et al., 2010; Sinclair et al., 2012; Munné-Bosch, 2015). Barrett et al. (2010) reported that most plant species exhibit equal or male-biased sex ratios, whereas female-biased sex ratios occur less frequently. Many researchers speculate that this phenomenon is most likely to be associated with the fundamental biological processes of gender dimorphism (Tognetti, 2012). However, we cannot ignore the fact that females have a higher resource requirement for reproduction than males. Obeso (2002) suggested that females invest more resources in reproduction, which would leave them with a smaller amount of resources available for defense. Fujita et al. (2014) suggested that if plants in phosphorus-limited communities invest little in sexual reproduction, plants would be in danger. In addition, sex-specific physiological responses, such as nitrogen uptake and use, would have a great effect on plant stress tolerance. For example, *Juniperus thurifera* females may use a long-term strategy by increasing N storage to compensate for massive reproductive masting events, whereas males prefer to use current nutrients for promoting gas exchange capacity (Montesinos et al., 2012). If plants grow in nutrient-poor conditions, especially under N shortage, the contrasting responses of males and females may have a direct impact on plant survival.

In *Populus*, a genus with male-biased sex ratios, females usually experience greater negative effects than do males under environmental stresses. To interpret this important case, a series of studies were carried out. The results suggested that different-sex individuals display distinct morphological and physiological adaptations to environmental stresses (Juvany and Munné-Bosch, 2015). For example, in a water-limited scenario, drought stress could limit poplars' photosynthetic capacity more in females than in males (Xu et al., 2008a). In addition, when the two sexes grow in a combined stress condition, such as elevated temperature (Xu et al., 2008b) or salinity (Chen et al., 2010), some sex-specific reactions will differ from single-stress responses. For instance, the combination of drought and salinity induced greater sex-specific differences in the levels of total chlorophyll, carotenoid, H_2_O_2_ and Cl^−^ in the leaves than did single stresses (Zhang et al., 2011). Furthermore, when poplars were subjected to low-temperature conditions, males exhibited improved chloroplast structure and more intact plasma membranes than did females, suggesting a better self-protective capacity (Zhang et al., 2011). *Populus* and *Salix*, belonging to Salicaceae, display different biased sex ratios that female-biased sex ratios are of approximately 2:1 in many willow species (Alliende and Harper, 1989; Dudley, 2006; Myers-Smith and Hik, 2012). Do female willows have a greater tolerance than males and thus survive multiply stressful conditions? So far, few studies have been performed to confirm the sex-specific mechanisms that produced female-biased sex ratios of willows in nature from an ecophysiological perspective (Jones et al., 1999; Dudley, 2006; Randriamanana et al., 2015). Moreover, previous studies on sexual differences in plant growth were merely based on a single time point, which may miss important information and could not reflect the temporal variation because sexes differ in their timing of development (Glynn et al., 2007; Hultine et al., 2013).

In this study, we employed *Salix paraplesia* individuals, which were grown with high and low soil fertility for about 140 days over one growing season. Briefly, our study monitored plants' growth parameters at three important time points, which respectively represent the initial-growth stage, rapid-growth stage and late-growth stage according to our observations for many years. The experiment was designed not only for research on how *S. paraplesia* males and females respond to nutrient limitation but also to provide support for research on willows' tolerance to low nutrients in nature. The latter is important because sporadic desertification has been observed in many of their natural habitats. Temporal variation in the relative growth rate (RGR), net assimilation rate (NAR), stable isotope compositions, non-structural carbohydrates (NSCs), and condensed tannins (CTs) were measured. It is hypothesized that there is a gender-specific trade-off between growth and defense, whereby females have a stronger capacity to overdraw on available resources to satisfy growth requirements (for example, greater biomass accumulation and higher RGR) or defense (for example, higher CT concentrations) than males under nutrient-poor conditions.

## Materials and methods

### Plant material and experimental design

In this study, 60 male and 60 female cuttings were produced from ten individuals of each population (6 populations in total) in their natural habitat in northern Sichuan province, China (Table 1). Briefly, each stem was cut into similar sections (1-cm diameter × 15-cm long) that were planted in 10-L plastic pots (one plant per pot), filled with 8 kg of homogenized soil on April 20. The two soil levels represented topsoil and deep soil. Topsoil was sieved from surface humus (0–15 cm), and deep soil was sieved from a depth of 20–40 cm at the site of origin. Topsoil represented nutrient-rich soil, and deep soil represented nutrient-poor soil. The properties of the nutrient-rich soil (nutrient-poor soil) used in this study were as follows (based on kg^−1^ dry soil): total carbon 30.33 g (17.86 g), organic carbon 28.51 g (16.08 g), total N 3.11 g (1.94 g), nitrate-N 26.06 mg (11.28 mg), and ammonium-N 21.86 mg (12.78 mg). The experimental layout was completely randomized with two factors (sex and nutrients). Local soil has high fertility; therefore, in the present study, topsoil with high fertility was used as the control soil. There were finally four treatments: (i) males with high soil fertility (control); (ii) females with high soil fertility (control); (iii) males with low soil fertility; and (iv) females with low soil fertility. Thirty plants of each sex were exposed to each treatment. To monitor the ontogenetic variation in plants responding to relative high and low soil fertility, plants were destructively harvested at 100 (representing initial-growth stage, on July 29), 120 (representing rapid-growth stage, on August 18), and 140 days (representing late-growth stage, on September 7) after plants were planted.

**Table 1:** **Willow population location and climate characteristics**.

**Population site name**	**Location**	**Elevation (m)**	**MAT (°C)**	**January MAT (°C)**	**July MAT (°C)**	**MAP (mm)**
Baxi	33°36′ N, 103°13′ E	3140	0.7	−10.6	10.8	656.8
Hongxing	34°05′ N, 102°44′ E	3150	1.1	−8.9	12.2	648.5
Rangtang	32°16′ N, 100°58′ E	3390	4.8	−2.7	14.1	763.0
Aba	32°54′ N, 101°42′ E	3400	3.3	−7.9	11.7	712.0
Hongyuan	32°47′ N, 102°32′ E	3485	1.1	−10.3	10.9	753.0
Dazhasi^*^	33°34′ N, 102°57′ E	3439	1.7	−9.4	11.5	660.0

* Dazhasi, study site. MAT, mean annual temperature; MAP, mean annual precipitation.

### RGR, NAR, and biomass allocation

Parameters related to plant growth were measured according to Glynn et al. (2007) with a minor modification. Briefly, leaf area was measured from the whole fully expanded leaves from each plant using a scanner with leaf area analysis software (WINFOLIA; Regent Instruments Inc. Quebec, Canada), after which leaves were dried at 60°C for 48 h and weighed. Leaf mass per unit area (LMA, g m^−2^) was then calculated as the quotient of the mass and area of the leaf sample. At harvest, plants were partitioned into leaf, stem and root fractions, respectively. Each plant fraction was dried at 60 °C for 48 h and weighed. Indices of plant growth and allocation were then calculated from dry mass and total leaf area measurements according to the following equations:

Total plant mass (g) = total leaf mass + total stem mass + total root mass;
RGR (g g^−1^ d^−1^) = [ln(final total mass) − ln(initial total mass)]/time;
Total leaf area (m^2^) = total leaf mass/LMA;
LAR (cm^2^ g^−1^) = total leaf area/total plant mass (LAR, leaf area ratio);
NAR (g m^−2^ d^−1^) = RGR/LAR;
SWR (g g^−1^) = stem mass/total plant mass (SWR, stem weight ratio);
RWR (g g^−1^) = root mass/total plant mass (RWR, root weight ratio).

### Carbon, nitrogen concentrations, and stable isotope signatures

The samples of shoots and roots were ground and passed through a 20-mesh screen after being dried (60°C, 48 h) to constant weight. The carbon and nitrogen concentrations were determined by the semi-micro Kjeldahl method as described by Kost and Boerner (1985), respectively. The stable carbon and nitrogen isotope abundance in the combusted samples was measured with a mass spectrometer (Finnegan MAT Delta-E, Bremen, Germany). The overall precision of the δ-values was better than 0.1‰, as determined from repeated samples.

### Condensed tannins

Condensed tannins (CTs) were analyzed using standard techniques (Orians, 1995; Hagerman and Butler, 1998; Hunter and Forkner, 1999). Briefly, leaves were removed from the trees and immediately put into a cooler, transported to the lab, and immediately vacuum dried. Once dry, leaves were ground with a mortar and pestle. Approximately 10 mg of leaf powder was weighed into 2-ml microfuge vials and washed with 500 μl diethyl ether. Following centrifugation (4 min at 3700 rpm), the diethyl ether was discarded. Tannins were extracted four times with 200 μl of a 7:3 acetone: water combination with 1-mM ascorbate. After each addition, samples were sonicated for 10 min at 5°C and centrifuged at 3700 rpm for 4 min. The supernatant was decanted into another microfuge vial. The acetone in the final supernatant was removed by evaporation. Water was added to attain the final volume of 500 μl. Some *S. paraplesia* tannins were prepared as standards in a similar manner, which generated by multiple sequential washes of samples in diethyl ether, followed by acetone extraction. *S. paraplesia* tannin standards were purified by Sephadex LH20 column chromatography. Samples were then analyzed using the butanol-HCl assay (Porter et al., 1985). Finally, tannin concentration (mg g^−1^ dry weight) was calculated.

### Non-structural carbohydrates

The NSC was determined as described by O'Brien et al. (2014). Five cuttings of each sex from each treatment were used to qualify the changes in stored NSC concentrations in the leaves, stems and roots, respectively. Plant materials were ground, and 15 mg of each sample was used for NSC analysis. Soluble sugars were extracted with a shaking bath of 80% ethanol at 27°C for one night, followed by two additional 2-h baths (Marquis et al., 1997; Myers and Kitajima, 2007). The remaining starch was digested with amyloglucosidase. The concentrations of soluble sugars and starch (measured as glucose equivalents) were measured at 487 nm by spectrophotometry after a phenol–sulphuric acid reaction. Mean NSC concentration was calculated by multiplying the extracted concentration for each organ by organ biomass, which provides total NSC per organ. The NSC values were summed and divided by total plant mass. Therefore, the NSC concentration in each organ represents the percentage of NSC relative to the whole cutting, and the sum of each organ percentage represents the total NSC concentration in the cutting.

### Statistical analyses

Experimental data were analyzed using SPSS 17.0 software (SPSS Inc., Chicago, IL, USA). Two-way analyses of variance were performed to evaluate the interaction effect of sex and variable nutrient conditions. Sexual differences were analyzed using a model with nutrient and sex as fixed effects. Significant individual differences among means of different treatments were determined by Tukey's multiple range tests after conducting tests of homogeneity for variances. Differences were considered as statistically significant at the *P* < 0.05 level.

## Results

### Plant growth

The treatments of nutrient availability had different effects on the plant growth of male and female *S. paraplesia* individuals (Tables 2, 3). Distinct sexual differences were detected in both total plant mass and total leaf area throughout the growing season. Briefly, nutrient limitation significantly decreased the accumulation of total plant mass of both sexes, and importantly, females consistently had relatively higher total plant mass than males. The effects of both sex and nutrient were significant for total plant mass on all three harvest dates. Similar results were observed for total leaf area with one exception: the nutrient factor had no effect for plants grown in the first harvest intervals (Days 1–100). However, sexually different LMA of both sexes were not detected on any of the three harvest dates, as confirmed by the statistical analyses (Table 3). Moreover, nutrient limitation had no effect on the LAR of either sex at the first harvest time (Day 100), but it did have obvious effects for males during the last two harvest intervals (Days 101–120, 121–140). Statistical analyses revealed a significant effect on LAR by nutrient. In addition, we found effects on SWR for sex, nutrient and their combination. These effects were significant when plants grown during the first (Days 1–100) and the last (Days 121–140) harvest intervals. However, there was no effect on SWR (except for the nutrient effect) for plants grown in the middle harvest interval (Days 101–120). There was an effect on RWR of nutrient for plants grown in the first two harvest dates, but this had no effect during the last harvest interval (Days 121–140). Interestingly, the effect of sex exhibited completely opposite changes, which could be associated with variation of the combined effects by sex × nutrient. Nevertheless, it should be noted that little interactive effect on plant growth by sex × nutrient was detected in most harvest intervals (Tables 2, 3). Although the interaction effect by sex × nutrient had no effect on RGR or NAR of males and females on any of the three harvest dates, distinct sexual differences existed for plants grown during the first two harvest intervals (Table 4). Over this time period, nutrient limitation significantly decreased cuttings' RGR and NAR when compared with those of cuttings grown under nutrient-rich conditions. Furthermore, females maintained relatively higher RGR and NAR than males under both conditions during the first harvest interval (Days 1–100). Statistical analyses suggested a significant effect on RGR and NAR by sex, which played an important role mainly during the first harvest interval (Days 1–100).

**Table 2:** **Statistical significance of single and interactive effects of sex and nutrient on parameters related to plant biomass accumulation based on two-way ANOVA over three harvest intervals (day 1–100, 101–120, and 121–140, respectively) after cuttings planted (April 20, 2014)**.

**Conditions**		**Total plant mass (g)**			**Stem weight ratio (SWR; g g^−1^)**			**Root weight ratio (RWR; g g^−1^)**		
		**Day 100**	**Day 120**	**Day 140**	**Day 100**	**Day 120**	**Day 140**	**Day 100**	**Day 120**	**Day 140**
Nutrient-rich	Male	1.97 ± 0.03b	6.22 ± 0.32b	14.62 ± 0.49b	0.37 ± 0.008c	0.55 ± 0.012a	0.52 ± 0.007ab	0.18 ± 0.006a	0.17 ± 0.006a	0.25 ± 0.001b
	Female	2.80 ± 0.08a	7.25 ± 0.07a	16.02 ± 0.19a	0.40 ± 0.006b	0.54 ± 0.009a	0.53 ± 0.006b	0.17 ± 0.005 b	0.17 ± 0.003a	0.26 ± 0.001b
Nutrient-poor	Male	1.59 ± 0.08c	3.08 ± 0.23c	6.58 ± 0.21d	0.40 ± 0.005b	0.42 ± 0.021b	0.39 ± 0.016c	0.15 ± 0.008b	0.19 ± 0.008a	0.30 ± 0.006a
	Female	2.26 ± 0.1b	3.89 ± 0.10c	8.08 ± 0.25c	0.52 ± 0.003a	0.40 ± 0.005b	0.58 ± 0.009a	0.15 ± 0.007b	0.19 ± 0.009a	0.19 ± 0.001c
	*P:F_S_*	^***^	^**^	^**^	^***^	ns	^***^	ns	ns	^***^
	*P:F_N_*	^***^	^***^	^***^	^***^	^***^	^**^	^**^	^*^	ns
	*P:F_S_*×*F_N_*	ns	ns	ns	^***^	ns	^***^	ns	ns	^***^

Different letters represent statistical significances between treatments (mean ± SE, n = 5) at P < 0.05 according to Tukey's multiple range tests. F_S_, sex effect; F_N_, nutrient effect; F_S_×F_N_, the interactive effect of sex and nutrient.

*Significance values of the factorial analysis (ANOVA) for the effects are denoted as follows: ns, non-significant*;

* *P < 0.05*;

** *P < 0.01*;

*** *P < 0.001*.

**Table 3:** **Statistical significance of single and interactive effects of sex and nutrient on leaf traits based on two-way ANOVA over three harvest intervals (day 1–100, 101–120, and 121–140, respectively) after cuttings planted (April 20, 2014)**.

**Conditions**		**Total leaf area (LA; cm^2^ plant^−1^)**			**Leaf mass per unit area (LMA; g cm^−2^)**			**Leaf area ratio (LAR; cm^2^ g^−1^)**		
		**Day 100**	**Day 120**	**Day 140**	**Day 100**	**Day 120**	**Day 140**	**Day 100**	**Day 120**	**Day 140**
Nutrient-rich	Male	104.28 ± 14.87ab	234.73 ± 21.12ab	372.15 ± 36.51a	83.79 ± 4.21a	74.46 ± 0.23a	89.89 ± 8.33a	52.79 ± 6.95a	37.57 ± 1.41c	25.42 ± 2.20b
	Female	145.78 ± 9.07a	289.11 ± 24.43a	347.67 ± 38.68a	83.56 ± 2.59a	73.16 ± 3.42a	101.77 ± 9.35a	51.96 ± 1.89a	39.83 ± 3.06bc	21.69 ± 2.36b
Nutrient-poor	Male	96.92 ± 4.16b	171.56 ± 12.58b	240.61 ± 12.53a	73.06 ± 2.88a	69.06 ± 3.58a	84.78 ± 1.17a	61.26 ± 2.68a	56.02 ± 4.64a	36.62 ± 2.01a
	Female	111.41 ± 11.71ab	204.46 ± 7.19b	242.51 ± 26.59a	67.97 ± 7.59a	78.09 ± 1.61a	80.58 ± 5.01a	49.21 ± 3.69a	52.58 ± 1.41ab	29.96 ± 2.71ab
	*P:F_S_*	^*^	^*^	ns	ns	ns	ns	ns	ns	ns
	*P:F_N_*	ns	^**^	^**^	^*^	ns	ns	ns	^**^	^**^
	*P:F_S_*×*F_N_*	ns	ns	ns	ns	ns	ns	ns	ns	ns

*Different letters represent statistical significances between treatments (mean ± SE, n = 5) at P < 0.05 according to Tukey's multiple range tests. F_S_, sex effect; F_N_, nutrient effect; F_S_×F_N_, the interactive effect of sex and nutrient*.

*Significance values of the factorial analysis (ANOVA) for the effects are denoted as follows: ns, non-significant*;

* *P < 0.05*;

** *P < 0.01*.

**Table 4:** **Statistical significance of single and interactive effects of sex and nutrient on growth parameters based on two-way ANOVA over three harvest intervals (day 1–100, 101–120, and 121–140, respectively) after cuttings planted (April 20, 2014)**.

**Conditions**		**Relative growth rate (RGR; g g^−1^ d^−1^)**			**Net assimilation rate (NAR; g cm^−2^ d^−1^)**		
		**Day 1–100**	**Day 101–120**	**Day 121–140**	**Day 1–100**	**Day 101–120**	**Day 121–140**
Nutrient-rich	Male	0.007 ± 0.0002b	0.057 ± 0.0019a	0.043 ± 0.0035a	1.327 ± 0.169b	15.296 ± 0.104a	17.174 ± 2.317a
	Female	0.01 ± 0.0003a	0.048 ± 0.0019a	0.04 ± 0.001a	1.982 ± 0.023a	12.065 ± 0.833b	18.781 ± 2.392a
Nutrient-poor	Male	0.005 ± 0.0005c	0.033 ± 0.0017b	0.038 ± 0.0046a	0.756 ± 0.105c	5.999 ± 0.626c	10.594 ± 1.767a
	Female	0.008 ± 0.0005b	0.027 ± 0.0036b	0.037 ± 0.0027a	1.662 ± 0.113ab	5.207 ± 0.723c	12.302 ± 0.958a
	*P:F_S_*	^***^	ns	ns	^***^	^*^	ns
	*P:F_N_*	^***^	^***^	ns	^**^	^***^	^*^
	*P:F_S_*×*F_N_*	ns	ns	ns	ns	ns	ns

Different letters represent statistical significances between treatments (mean ± SE, n = 5) at P < 0.05 according to Tukey's multiple range tests. F_S_, sex effect; F_N_, nutrient effect; F_S_×F_N_, the interactive effect of sex and nutrient.

*Significance values of the factorial analysis (ANOVA) for the effects are denoted as follows: ns, non-significant*;

* *P < 0.05*;

** *P < 0.01*;

*** *P < 0.001*.

### Carbon, nitrogen concentrations and carbon, nitrogen isotope signatures

Multiple effects (sex, nutrient, and their combination) were generally not significant for C concentration, N concentration, C/N ratio or δ^13^C in the shoots and roots of *S. paraplesia* male and female cuttings (Table 5). Nutrient limitation significantly increased δ^15^N in shoots, and females had more δ^15^N than did males. Statistical analyses suggested a significant effect on the δ^15^N in shoots by sex and by nutrient, but the interaction effect by sex × nutrient was not significant. In addition, nutrient limitation increased δ^15^N in roots, but the changes were only detected in males (Table 5). Statistical analyses indicated that the effect on δ^15^N by nutrient played a key role in roots, but the effect by sex as well as the interaction effect by sex × nutrient was not significant (Table 5).

**Table 5:** **Statistical significance of single and interactive effects of sex and nutrient on carbon (C), nitrogen (N) concentrations, C/N ratios, carbon and nitrogen isotope composition of shoots and roots based on two-way ANOVA at the last harvest time point (Day 140)**.

**Conditions**		**C (g kg^−1^)**		**N (g kg^−1^)**		**C/N ratio**		**δ^13^C (‰)**		**δ^15^N (‰)**	
		**Shoot**	**Root**	**Shoot**	**Root**	**Shoot**	**Root**	**Shoot**	**Root**	**Shoot**	**Root**
Nutrient-rich	Male	473.82 ± 4.44a	407.1 ± 8.81a	26.46 ± 0.84a	20.65 ± 0.85a	17.95 ± 0.73a	19.75 ± 0.44a	−26.33±0.2a	−25.69±0.16a	1.8 ± 0.11c	3.86 ± 0.26b
	Female	472.56 ± 0.91a	419.49 ± 6.88a	28.62 ± 1.36a	19.11 ± 0.36a	16.59 ± 0.79	21.98 ± 0.72a	−26.43±0.14a	−25.83±0.09a	2.32 ± 0.07b	3.83 ± 0.1b
Nutrient-poor	Male	463.14 ± 1.38a	397.38 ± 10.63a	28.52 ± 0.73a	20.23 ± 0.46a	16.26 ± 0.41a	19.68 ± 0.92a	−26.76±0.27a	−25.45±0.31a	2.36 ± 0.11b	4.93 ± 0.27a
	Female	473.44 ± 2.83a	410.72 ± 7.77a	27.74 ± 0.24a	20.94 ± 0.57a	17.07 ± 0.24a	19.63 ± 0.45a	−26.53±0.12a	−25.68±0.16a	2.9 ± 0.13a	4.33 ± 0.11ab
	*P:F_S_*	ns	ns	ns	ns	ns	ns	ns	ns	^**^	ns
	*P:F_N_*	ns	ns	ns	ns	ns	ns	ns	ns	^**^	^**^
	*P:F_S_*×*F_N_*	ns	ns	ns	ns	ns	ns	ns	ns	ns	ns

*Different letters represent statistical significances between treatments (mean ± SE, n = 5) at P < 0.05 according to Tukey's multiple range tests. F_S_, sex effect; F_N_, nutrient effect; F_S_×F_N_, the interactive effect of sex and nutrient. Significance values of the factorial analysis (ANOVA) for the effects are denoted as follows: ns, non-significant*;

** *P < 0.01*.

### Non-structural carbohydrates and condensed tannins

**Figure 1:**
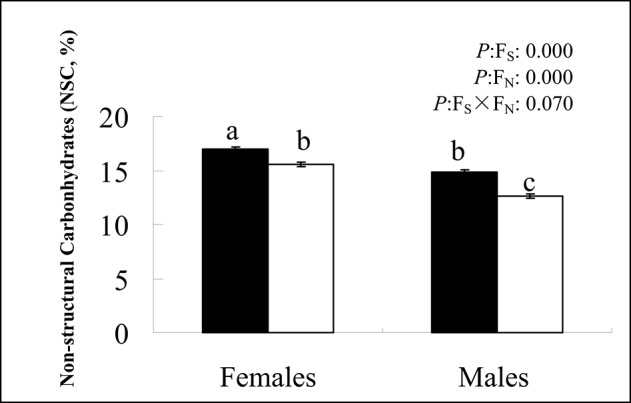
**Effects of nutrient availability on non-structural carbohydrates (mean ± SE, *n* = 5) in *Salix paraplesia* males and females**. Nutrient-poor condition: black; nutrient-rich condition: white. Different letters above the bars represent statistically significant differences between treatments at *P* < 0.05 according to Tukey's multiple range tests. Significance values of the factorial analysis (ANOVA) are denoted as follows: sex, sex effect; nutrient, nutrient effect; sex × nutrient, sex × nutrient interaction effects.

Low soil fertility increased plants' NSC levels, but the increases were different in males and females. Briefly, females had more NSC than did males (Figure 1). Statistical analyses suggested an effect on the NSC by sex and by nutrient, but the interaction effect by sex × nutrient was not significant. In addition, there was a positive relationship between total plant mass and NSC. Females not only accumulated more NSC but also had greater total plant mass than males. Table 6 revealed that nutrient limitation caused plants to produce more CTs among females than males on Day 100. This effect gradually diminished toward the end of the growing season. An effect on the CTs by nutrient was found throughout the plant growing season, while the effect by sex was only detected on Day 100. Females possessed more CTs than did males under nutrient-poor conditions on Day 100, but this pattern was completely changed when plants were subjected to nutrient-rich conditions. However, the sexual differences in CT concentration between males and females detected on Day 120 or 140 were not significant (except plants under nutrient-poor conditions on Day 140).

**Table 6:** **Statistical significance of single and interactive effects of sex and nutrient on condensed tannins (CTs) based on two-way ANOVA over three harvest intervals (day 1–100, 101–120, and 121–140, respectively) after cuttings planted (April 20, 2014)**.

**Conditions**		**Condensed tannins (CTs, mg g**^−1^ **dry weight)**		
		**Day 100**	**Day 120**	**Day 140**
Nutrient-rich	Male	2.309 ± 0.06c	0.535 ± 0.06b	0.261 ± 0.03b
	Female	1.507 ± 0.05d	0.52 ± 0.03b	0.222 ± 0.03b
Nutrient-poor	Male	2.993 ± 0.09b	1.399 ± 0.1a	0.357 ± 0.02b
	Female	3.37 ± 0.07a	1.11 ± 0.06a	0.533 ± 0.05a
	*P:F_S_*	^*^	ns	Ns
	*P:F_N_*	^***^	^***^	^***^
	*P:F_S_*×*F_N_*	^***^	ns	^*^

*Different letters represent statistical significances between treatments (mean ± SE, n = 5) at P < 0.05 according to Tukey's multiple range tests. F_S_, sex effect; F_N_, nutrient effect; F_S_×F_N_, the interactive effect of sex and nutrient. Significance values of the factorial analysis (ANOVA) for the effects are denoted as follows: ns, non-significant*;

* *P < 0.05*;

*** *P < 0.001*.

## Discussion

Male and female plants play different roles in productive biology and thus have sexually different resource demands imposed upon them. Any decision about resource utilization made by males or females may have different consequences, even contributing to spatial segregation of the sexes (Fujita et al., 2014). Many studies have implied that the population sex ratio of certain species would become female-biased if dioecious plants were placed under high-quality environments. The same reports maintain that the sex ratio would become male biased under stressful or resource-poor habitats, because reproduction is very costly for females (Sanchez-Vilas et al., 2012). However, there are exceptions. *Salix* often exhibits female-biased population sex ratios in naturally stressful environments (Dudley, 2006; Myers-Smith and Hik, 2012). Thus, we have attempted to find possible explanations that gender-specific trade-offs between growth and defense may be relevant to willow's female-biased sex ratios. In this study, *S. paraplesia* male and female cuttings were employed. A critical subject is posed: once plants suffer from nutrient limitation, how do males and females make their own choices: growing fast enough to compete or maintaining physiological adaptations necessary for survival?

Estimating growth parameters can help to elucidate whether plants suit the current environment or not. In the present study, female cuttings are likely to accumulate greater biomass than males under either nutrient-rich or nutrient-poor conditions. Statistical analyses have indicated that both sex and nutrients are important factors. Hermans et al. (2006) indicated that plants often allocate biomass to the root system when mineral elements are scarce. However, in our study, nutrient limitation significantly decreased biomass allocation below ground (see RWR) in males, while few effects were observed in females compared with those under nutrient-rich conditions during the initial-growth stage. This suggests that nutrient-limitation did not cause severe effects on the root growth of female cuttings but rather was fatal to males. This may ultimately reduce male cuttings' survival rate and might explain the segregation of the sexes. The largest value of RWR was observed in males under nutrient-poor conditions during the late-growth stage, suggesting that males indeed sensed and experienced greater environmental stress than did females. In other words, nutrient limitation may cause more severe impacts on plant growth in males than in females. In addition to RWR, nutrient limitation increased resource output to the stem in both sexes, and females constantly had higher SWR than males. *S. paraplesia* females gave priority to vegetative investment at the cost of stem growth during the initial- and late-growth stages, which may have enhanced stem functions including water transport and mechanical stability (Albrectsen et al., 2004; Taneda and Tateno, 2004). Relatively higher levels of nutrient storage in the female cuttings used for propagation may partially explain why female cuttings exhibited stronger capacity than male cuttings during the initial-growth stage.

In addition to plant growth, two compounds' NSC and CTs were investigated. NSC stores are assumed to be an important trait for plant survival under stress (O'Brien et al., 2014), particularly in maintaining basic metabolic functions to optimize growth and defense (Dietze et al., 2014). In general, most carbohydrates are produced in foliage leaves, and some are synthesized in flowers and fruits (Kozlowski, 1992). However, trees in the juvenile stage of development do not flower and, therefore, only have vegetative tissues as carbohydrate sinks. In our study, we analyzed the total NSC contents of entire cuttings (Figure 1). As expected, low soil fertility increased plants' NSC content, conducive to enhancing plant tolerance capacity (Chapin et al., 1990). Interestingly, females had higher NSC levels than males, and statistical analyses indicated a role for sex. This suggests that female cuttings with greater NSC storage were more likely to survive nutrient-poor conditions (Canham et al., 1999). In addition, there was a positive relationship between total plant mass and NSC, suggesting a good balance between photosynthesis and respiration, which ultimately influences carbon availability for growth (Chapin et al., 1990; Dietze et al., 2014).

Recent advances in plant metabolism studies indicate that plants are dependent on the deployment of secondary metabolites for their response to abiotic and biotic stresses (Mumm and Hilker, 2006; Neilson et al., 2013), and a variety of secondary compounds are produced from carbohydrates, amino acids and lipids. For example, *Populus tremuloides* seedlings increased CT concentrations under conditions of low fertility with competition (Donaldson et al., 2006). In the present study, comparative analysis showed that females produced more CTs than males under nutrient-poor conditions, while producing fewer CTs than males when plants are subjected to nutrient-rich conditions during the initial-growth stage. This suggests that females are likely more sensitive than males to nutrient availability, with regard to chemical defense. Our studies indicate that CTs may play a different role, sexually, in the initial- and late-growth stages rather than the rapid-growth stage (Table 6), which could provide females with greater defense capacity than enjoyed by males. In contrast, CTs are traditionally thought to play a key role in plant defense against herbivorous insects (Agrawal, 2007; Gols, 2014; Agrawal and Weber, 2015). In our study, temporal variation and sex-based differences in the regulatory strategies of CTs were observed in males and females. This may suggest sex-based differences between willows and herbivorous insects and sex-based differences in competition- and resource-mediated trade-offs between growth and defense (Donaldson et al., 2006).

According to the growth-differentiation balance hypothesis (Herms and Mattson, 1992; Glynn et al., 2007), if levels of defense remain stable while resources decrease, fewer resources will be available for growth or reproduction. Stamp (2003) suggested that resources allocated to the synthesis, storage, and regulation of plant secondary metabolites would come at the expense of those allocated for growth and reproduction. In accordance with our hypotheses, the results presented here suggest sex-specific trade-offs between the growth and defense of willows facing nutrient limitation. In the initial-growth stage, male and female strategies when confronting challenges related to nutrient availability differ substantially. Under nutrient-rich conditions, males invested more resources in defense than females (as reflected in the synthesis of more condensed tannins than females, see Table 6), which could be defined as a conservative adaptive strategy. This could impact male cuttings' growth (Table 4). Although high resource availability may diminish allocation costs and allow for growth and defense (Siemens et al., 2002), once plants face limited resources, females' positive regulatory mechanisms seem to be more efficient than those of males. It was found that females synthesized more CTs to enhance defense; levels were greater than those found in males (Table 6). As predicted, nutrient limitation significantly decreased plants' RGR and total plant mass accumulation, but females still had higher RGR and greater total plant mass than males (Table 4). Most explanations of plant stress tolerance indicate an evolutionary trade-off such that plant species with high chemical defense would lower their RGR to maintain relative equilibrium (Herms and Mattson, 1992; Stamp, 2003; Fine et al., 2006; Glynn et al., 2007). However, in our study, females were likely to adopt radical strategies, overdrawing on available resources to satisfy both growth and defense, suggesting sex-based strategy differences. The results also indicated that females may have an extra mechanism to compensate for the investment in growth under nutrient-poor conditions. For example, female *Salix* may have higher photosynthetic rate than males, or increase allocation to photosynthetic organs, which would enable them to compensate for their higher resource demands (Dudley and Galen, 2007; Ueno et al., 2007). Similar relationships between RGR and CTs were observed when males and females were in the late-growth stage. These results suggest that such aggressive mechanisms could help female individuals win from the start when willows face intersexual competition for limited resources. In the rapid-growth stage, both males and females maintained a high level of growth, and CT levels were significantly decreased when compared with those measured in the initial-growth stage, suggesting an obvious trade-off between growth and defense (Stamp, 2003). The total biomass of females was found to be significantly higher than that of males under nutrient-rich conditions, but nutrient shortages could alter this relationship (the differences between sexes were not substantial). Previous studies have suggested that biomass allocation plays a key role in the plant response to nutrient shortage (Hermans et al., 2006; Agrawal, 2007), and we speculate that different strategies for biomass allocation may result in sex-based differences in defense capacity and trade-offs between growth and defense.

Recent studies have suggested various inter-sexual competition patterns in a male-biased dioecious plant *Populus* with a sex ratio, in terms of resource utilization within given environments (Chen et al., 2014, 2015). Our study further provided new insights into *S. paraplesia*, a female-biased sex ratio dioecious plant, in response to nutrient availability. In particular, we demonstrated sex-specific trade-offs between growth and defense. Females are likely to adopt radical strategies, overdrawing on available resources to satisfy both growth and defense, which seems to be more like a gamble compared to the strategies pursued by males. Females may have an extra mechanism to compensate for the investment in growth under nutrient-poor conditions.

## Author contributions

HJ designed the experiment and measured the dynamic changes of plant biomass; he was also responsible for the statistical analysis and manuscript writing. SZ contributed largely to language improvement, and provided much useful suggestions and comments for the revision. YL and GX carried out much of the field work and chemical analyses. DZ had the initial research idea and was also doing some of the writing of the paper during initial submission and revisions.

### Conflict of interest statement

The authors declare that the research was conducted in the absence of any commercial or financial relationships that could be construed as a potential conflict of interest.
